# Barium Titanate Functionalization with Organosilanes: Effect on Particle Compatibility and Permittivity in Nanocomposites

**DOI:** 10.3390/molecules27196499

**Published:** 2022-10-01

**Authors:** Nico Zamperlin, Andrea Bottacini, Emanuela Callone, Alessandro Pegoretti, Marco Fontana, Sandra Dirè

**Affiliations:** 1 Department of Industrial Engineering, University of Trento, Via Sommarive 9, 38123 Trento, Italy; 2 National Interuniversity Consortium of Materials Science and Technology (INSTM), Via G. Giusti, 9, 50121 Firenze, Italy; 3 Institute of Mechanical Intelligence, Scuola Superiore Sant’Anna, Piazza Martiri della Libertà 33, 56127 Pisa, Italy

**Keywords:** barium titanate, functionalization, organosilanes, interface, nanocomposites, dielectric constant

## Abstract

Barium titanate (BT) recently gained new interest in the preparation of dielectric and piezoelectric lead-free materials for applications in sensors, electronics, energy harvesting and storage fields. Barium titanate nanocomposites can achieve attractive performance, provided that the compatibility between ceramic particles and polymeric matrices is enhanced to the benefit of the physical properties of the final composite. Tuning the particle–matrix interface through particle functionalization represents a viable solution. In this work, surface functionalization of BT nanoparticles (NPs), obtained by hydrothermal synthesis, with 3-glycidyloxypropyltrimethoxysilane, 2-[(acetoxy(polyethyleneoxy)propyl]triethoxysilane and triethoxysilylpropoxy(polyethyleneoxy)dodecanoate, was performed after optimizing the hydroxylation process of the NPs to improve their surface reactivity and increase the yield of grafting. Solid-state nuclear magnetic resonance and thermogravimetric analysis were used to quantify the molecules grafted onto the ceramic nanoparticles. Both bare and functionalized particles were employed in the realization of epoxy- and polydimethylsiloxane (PDMS)-based nanocomposites. Functionalization was proven to be beneficial for particle dispersibility and effective for particle alignment in the PDMS matrix. Moreover, the dielectric constant measurements revealed the potential of PDMS-based nanocomposites for applications in the field of dielectric elastomers.

## 1. Introduction

Materials showing piezoelectric and dielectric properties find an increasing number of applications, and valid lead-free alternatives to lead-zirconate-titanate (PZT) have gained attention in the last few years [1]. Moreover, applications in microelectronics, energy storage, energy harvesting and flexible sensors are driving efforts toward engineering composite materials with enhanced piezoelectric and dielectric properties [2,3,4]. Barium titanate (BT), which was the most used material before the appearance of PZT [5], in this sense has been better investigated in the last few decades as active ceramic component of such materials, from the point of view of both its synthesis and dispersion capability in polymeric matrices. Nanometric particles (NPs) produced following different routes with respect to traditional calcination offer incredible opportunities to develop new, high-performing materials with reduced energy consumption [6,7,8]. Matrices of interest in the production of BT/polymer nanocomposites are epoxy resins and silicone elastomers: BaTiO_3_/epoxy systems have been extensively studied and present many advantages for embedded capacitor films, such as low cost, low dielectric loss, low conductivity and leakage current [9,10]. Polydimethylsiloxane (PDMS) is instead a popular choice in the design of piezoelectric composite nanogenerators in flexible devices and wearable applications [2,3]. Studies on these systems have evidenced their enhanced properties due to the anisotropic alignment of BT∙NPs along a preferential direction [11,12], similar to what has been seen in PLA composites with aligned BT nanowires [13].

NPs have high values of specific surface area, making them more reactive than larger particles. In fact, they have a strong tendency to aggregate to reduce surface energy, affecting homogeneity and acting as defect sites in nanocomposite materials. They also distort the local electric field and weaken the breakdown strength when subjected to an electric field [14,15]. If the breakdown strength decreases due to the presence of aggregates, the maximum electric field to carry out the poling (alignment of crystallites’ dipole moments) is decreased, worsening the net polarization, dielectric and piezoelectric properties of the composite. The interaction between particles and the surrounding polymeric matrix is the key to control the phenomenon. The lower the filler-matrix compatibility, the higher the aggregation: polar, hydrophilic particles tend to minimize the interfacial area with a non-polar polymeric matrix and will form aggregates. Accordingly, NPs’ surfaces are typically chemically modified to improve the compatibility with the organic matrix and achieve uniform dispersion and optimal properties [16]. Improving particles’ dispersibility through functionalization allows one to increase the filler loading in the nanocomposite, with better control of the formation of aggregates compared to bare particles [17], and positive effects on dielectric and piezoelectric properties. The functionalization of metal oxide NPs with organosilane coupling agents (grafting-to approach) is one of the most widely used methods [15,18], and the large availability of organosilanes with different organic functional groups permits us to modify both particle/matrix and particle/particle interactions. Organosilanes react with the surface hydroxyl groups (-OH) of the particles, but for poorly hydroxylated surfaces, as in the case of BT, the degree of functionalization can be insufficient. Hydroxylation with hydrogen peroxide has been shown to be effective in increasing the density of hydroxyl groups on barium titanate NPs by up to 16%, thus favoring the subsequent grafting of different functional groups [18,19,20].

We previously reported on the optimization of BT NP synthesis by the sol–gel route under hydrothermal conditions [21]. This work explores the potential of these particles in the production of polymer nanocomposites, focusing on the optimization of BT functionalization with different organosilanes and evaluating the effect of functionalization on particles’ dispersion and matrix–filler interactions in two different polymeric matrices. A preliminary study was performed on commercial BT particles to select the hydroxylation conditions that lead to the improved reactivity of NPs toward reaction with 3-glycidyloxypropyltrimethoxysilane (G). The optimized procedure was applied to the functionalization with G, 2-[(acetoxy(polyethyleneoxy)propyl]triethoxysilane (A) and triethoxysilylpropoxy(polyethyleneoxy)dodecanoate (T) of BT nanoparticles prepared by sol-gel reactions under hydrothermal conditions [21]. Thermogravimetric analysis (TGA), scanning electron microscopy (SEM) and solid-state nuclear magnetic resonance (NMR) were used to study both hydroxylation and functionalization steps. Fabrication of epoxy and polydimethylsiloxane-based nanocomposites with two different loadings of bare and functionalized NPs was carried out. This step was completed while applying a DC electric field during cross-linking of the polymeric matrix to try to induce the alignment of the BT nanoparticles’ dipole moments. Particles’ dispersion and particle/matrix interfaces were investigated by SEM and energy-dispersive X-ray spectroscopy (EDXS), and preliminary results on nanocomposites’ dielectric permittivity were also obtained.

## 2. Results and Discussion

### 2.1. Functionalization of BT Particles

**Figure 1:**
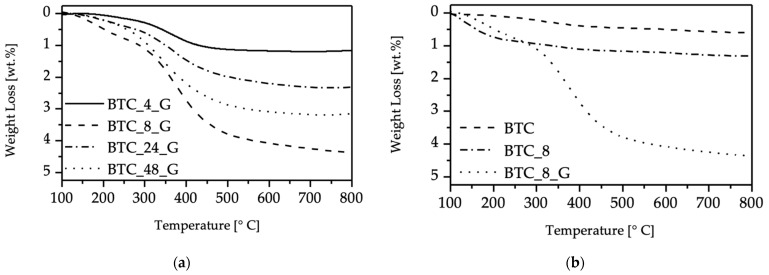
TGA analyses of (**a**) commercial BT particles hydroxylated with different reaction times and then functionalized; (**b**) TGA curves of pristine BTC sample, and the samples obtained with 8 h hydroxylation and functionalization. TGA plots of commercial particles are shown in the range 100–800 °C since there are no evident thermal phenomena below 100 °C.

The study of BT functionalization was done on commercial particles (BTC), which were reacted with H_2_O_2_ for different reaction times and then with 3-glycidyloxypropyltrimethoxysilane. To evaluate the effect of the hydroxylation step on functionalization, TGA analyses were carried out on functionalized BTC particles previously subjected to hydroxylation, respectively, for 4, 8, 24 and 48 h (Figure 1a). For all the samples, the first weight loss observed in the range 100–300 °C is due to adsorbed water and residual hydroxyl groups and appears more pronounced for BTC_8_G (8 h hydroxylation) and BTC_24_G samples (24 h hydroxylation). Figure 1b presents the TGA curves of the sample hydroxylated for 8 h (BTC_8) and subsequently functionalized (BTC_8_G). The comparison points out the reduction in the amount of residual -OH as a consequence of the reaction with the organosilane. The major weight loss in the range between 300 °C and 600 °C is due to the thermal decomposition of the organic groups introduced by organosilane grafting and depends on the hydroxylation time. The functionalization is not favored by hydroxylation times longer than 8 h; indeed, sample BTC_8_G presents the highest mass loss in the range 300–600 °C. These results can be explained by considering that, under the experimental conditions used for hydroxylation, condensation of -OH groups can be favored by prolonged treatments, thus reducing their availability for functionalization. The measured weight losses in the range 300–600 °C (as reported in Section 3.5) were used to calculate the content of organosilane grafted on BTC particles hydroxylated for different time (Table 1).

**Figure 2:**
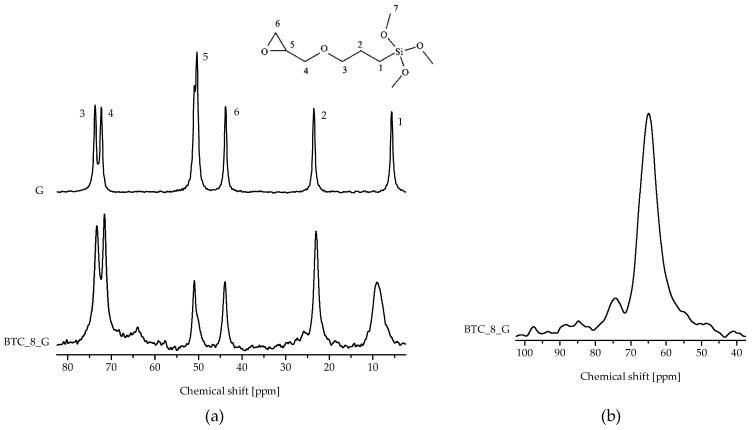
(**a**) ^13^C CPMAS NMR spectra of the neat G organosilane and of the BTC particles hydroxylated for 8 h and functionalized with the same silane; carbon atoms are labeled according to the scheme shown in the top of figure. (**b**) ^29^Si CPMAS NMR spectrum of the BTC_8 G sample.

Solid-state NMR was applied to study the degree of organosilane condensation, check the integrity of the glycidoxypropyl chain and evaluate the functionalization yield. Figure 2a shows the ^13^C CPMAS spectra of neat organosilane and the BTC_8_G sample; carbon resonances are assigned according to the scheme reported in the figure. All the signals belonging to the organosilane functional group are present in the spectrum of the BTC_8_G sample. In detail, the resonances of methylene carbons can be identified at 9 (1), 23 (2) and 73.1 (3, 4) ppm, respectively; signals due to methine (6) and methylene (5) carbons of the epoxy group are found at 43.11 and 50.9 ppm, respectively [22]. The -OCH_3_ resonance is not observed at around 50 ppm, indicating the complete organosilane hydrolysis. The broadness and downfield shift (with respect to the position for the pure reagent) of peak 1 indicate the occurrence of condensation, which could be due both to grafting onto the particles’ surface or organosilane self-condensation. Partial epoxy ring opening with the formation of diols is indicated by the reduced intensity of peaks 5 and 6 with respect to the others, the presence of a weak resonance at 64.8 ppm and the intensity of resonances 3 and 4. Similar results were achieved for the other samples (Figure S1, see Supplementary Materials).

Organosilane condensation is better proven by the ^29^Si CPMAS spectrum of the BTC_8_G sample (Figure 2b), which presents two resonances, respectively, assigned to T^2^ (at −56.5 ppm, representing SiCO_3_ units with two bridging oxygens) and T^3^ (66.0 ppm, SiCO_3_ fully condensed units) Si units, with ratio 7/93, as confirmed by the quantitative ^29^Si MAS experiment (not reported).

The amount of grafted organosilane in the BTC samples was calculated from ^13^C spectra according to the procedure described in Section 3.5, and the results are shown in Table 1.

The quantitative results of ^13^C NMR and TGA analyses are in good agreement and point out that running the hydroxylation reaction for 8 h leads to the highest functionalization degree of BT nanoparticles.

**Figure 3:**
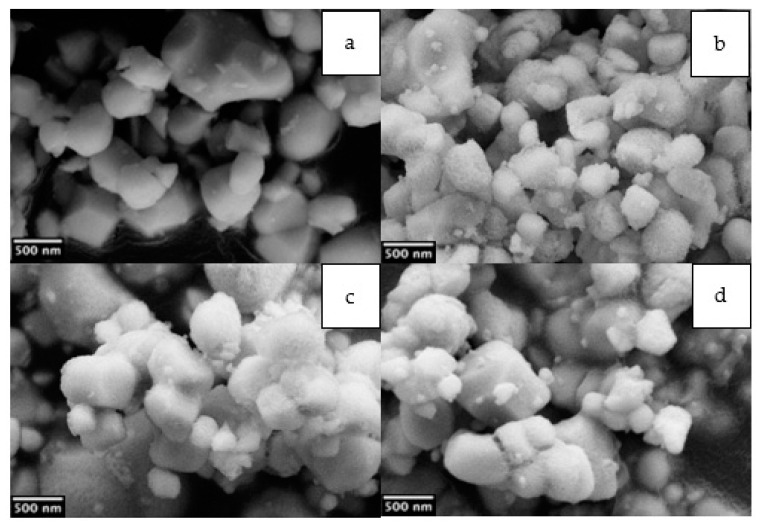
SEM images of the commercial BTC (**a**), and samples hydroxylated for 8, 24 and 48 h, BTC_8 (**b**), BTC_24 (**c**), BTC_48 (**d**).

Figure 3 shows the SEM images of pristine and hydroxylated nanoparticles, namely BTC, BTC_8, BTC_24 and BTC_48. The starting commercial nanoparticles (Figure 3a) possess an average diameter of 500 nm, with broad size distribution [21] and a limited degree of aggregation, which is enhanced by the hydroxylation step (Figure 3b), particularly for longer times (Figure 3c,d). Aggregation of the particles caused by prolonged hydroxylation justifies the lower functionalization degree detected by TGA and NMR. On the other hand, too short hydroxylation times are not able to introduce sufficient hydroxyl groups for the subsequent reaction. A hydroxylation step of 8 h was therefore selected for studying the functionalization with different organosilanes of sol-gel nanoparticles synthesized in hydrothermal conditions [21].

**Figure 4:**
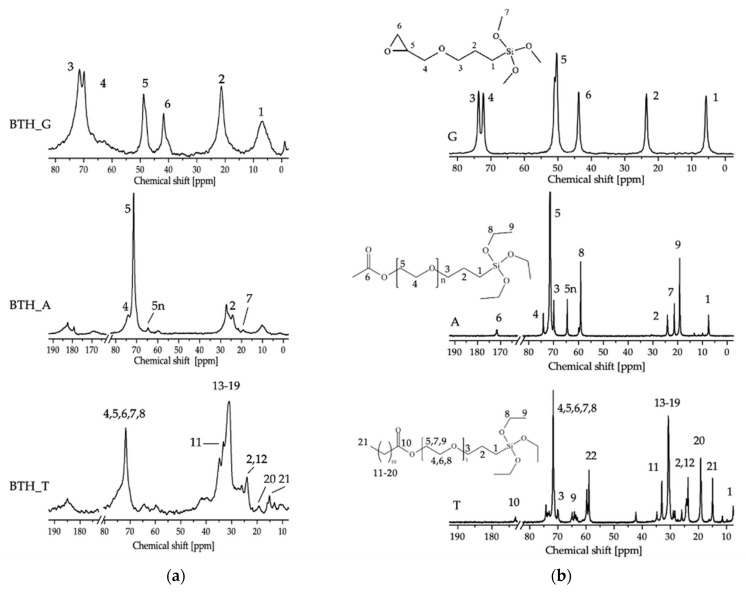
^13^C CPMAS NMR spectra of (**a**) BTH particles functionalized with 3-glycidyloxypropyltrimethoxysilane (BTH_G), 2-[(acetoxy(polyethyleneoxy)propyl]triethoxysilane (BTH_A) and triethoxysilylpropoxy (polyethyleneoxy)dodecanoate (BTH_T); (**b**) ^13^C NMR spectra of neat alkoxysilanes (the carbon atom labeling is shown in the schemes).

Figure 4a shows the ^13^C CPMAS NMR spectra of the particles obtained by hydrothermal synthesis (BTH), hydroxylated for 8 h and functionalized with 3-glycidyloxypropyltrimethoxysilane (G), 2-[(acetoxy(polyethyleneoxy)propyl]triethoxysilane (A) and triethoxysilylpropoxy (polyethyleneoxy)dodecanoate (T), respectively. The ^13^C NMR spectra of neat organosilanes are reported in Figure 4b for comparison, together with molecular structures and carbon labeling. All the signals of the corresponding organosilanes are present in the functionalized BTH nanoparticles, proving effective grafting. The BTH_G spectrum is similar to the one recorded on the BTC_8_G sample, but the peak intensity suggests an improvement in the functionalization favored by the higher surface area displayed by BTH particles, which present an average diameter of 120 nm [21].

In the case of BTH_A, the signal to noise ratio (S/N) is quite low as a consequence of the reduced amount of anchored silane in comparison with G-functionalized particles. This is likely due to the silane reactivity, which is negatively affected by both steric and electronic inductive effects of the long organic chain. The downfield shift at 10 ppm of resonance 1 indicates condensation, but residual ethoxy groups are still visible at 60 and 19 ppm. The C=O resonance is split into two signals at 183 and 169 ppm with a ratio of 82/18, probably as a consequence of different local environments experienced by the carbonyl groups in open or folded chains.

The spectrum of BTH_T deserves similar comments: the S/N is quite low, accounting for a low amount of anchored silane, and residual ethoxy groups (60 and 15 ppm) suggest incomplete hydrolysis.

The ^29^Si spectra of BTH_A and BTH_T samples (not shown) suffer from the low amount of Si in the whole sample, leading to a very low S/N. In both samples, a broad resonance due to the overlapping of T^2^ (at −55 ppm) and T^3^ (at −65 ppm) peaks, with a ratio T^2^/T^3^ of around 30/70, can be argued, indicating that the condensation degree is lower with respect to the case of G-functionalized particles.

Thermogravimetric analyses were carried out on the functionalized BTH particles to quantify the degree of grafting, obtaining TGA curves similar to those of functionalized BTC samples. For the BTH_G sample, the weight loss in the range 300–600 °C attributed to the G silane decomposition was 3.3 wt.%, thus confirming that the functionalization was enhanced with respect to the commercial BT particles (Table 1).

BTH particles functionalized with the A and T organosilanes present comparable weight losses, namely 3.0 and 2.5 wt.%, respectively. Considering the molecular weight of the three employed organosilanes, A and T lead to a more difficult grafting onto the surface of the particles with respect to G, as suggested by NMR. The degree of functionalization was evaluated according to Maitra et al. [23] by calculating the grafting density of the organosilanes σ, expressed as the number of grafted molecules per square nanometer (Section 3.5, Equation (1)). Resulting grafting densities are 10.6 molecules nm^−2^ for BTH_G, 3.5 molecules nm^−2^ for BTH_A and 3.2 molecules nm^−2^ for BTH_T. These values are in agreement with NMR considerations. It must be considered also that A and T not only have a longer organic chain with respect to G, but also present different alkoxy groups. The ethoxy group has, in general, lower reaction rates with respect to the methoxy group [18], and this may also play a role. Nevertheless, grafting of all the considered organosilanes is proven successful.

### 2.2. BT-Epoxy and BT-PDMS Nanocomposites

**Figure 5:**
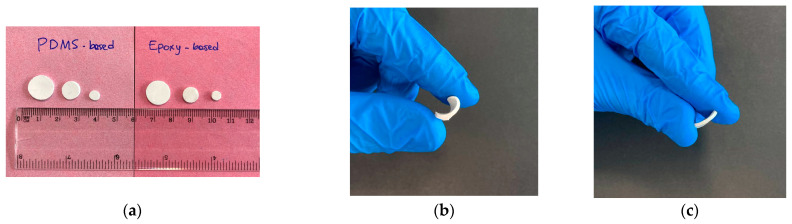
PDMS and epoxy-based composites produced in different sizes (**a**); PDMS-based composites (**b**) present higher flexibility than epoxy-based composites (**c**).

Functionalization of hydrothermally synthesized nanoparticles has been shown to produce a higher density of organosilane molecules on the particles’ surface with respect to commercial particles, thus making BTH more attractive for composite production. Accordingly, polymer-based nanocomposites were produced with both bare and G-functionalized BTH particles. PDMS- and epoxy-based composites (Figure 5) with 3.5% and 21% volumetric BTH_G content were produced to assess the effect of functionalization on the dispersibility of BT particles. To compare the effect of functionalization with different organosilanes, nanocomposites with BTH_A particles were prepared at 3.5 vol.% content. The samples were produced by casting the polymer mixtures containing both bare and modified nanoparticles and applying a combined poling/thermal curing process.

PDMS composites showed generally higher flexibility and a better appearance with respect to the epoxy ones, with smoother surfaces and a lower concentration of surface defects. However, the preparation of the PDMS sample loaded with bare BTH particles at 21 vol.% filler content was not totally successful, as the sample remained sticky even after a post-curing treatment. On the contrary, the same composition with G-functionalized BTH particles was successfully produced, proving the positive effect of particle functionalization for the achievement of high filler loading. Differential scanning calorimetry (DSC) analyses of composites (Table S1, see Supplementary Materials) demonstrated that, in general, the addition of the NPs does not lead to relevant changes in the thermal properties of the polymeric matrix. All PDMS-based samples present similar T_g_ values (120–125 °C). The faultiness of the sample pdms_21_BTH could be due to the non-optimized production process. Furthermore, a low filler concentration does not have remarkable effects on crystallization and melting enthalpies, but at a high filler concentration, we record lower values for melting and crystallization enthalpies. Concerning the epoxy-based samples, the glass transition temperature (T_g_) of epoxy samples is in the range 6–9 °C, except for the sample epoxy_21_BTH_G, which presents a T_g_ = 26 °C. In this case, the high loading of functionalized NPs provides a large number of oxirane rings available for the ring-opening reaction, improving the cross-linking degree.

**Figure 6:**
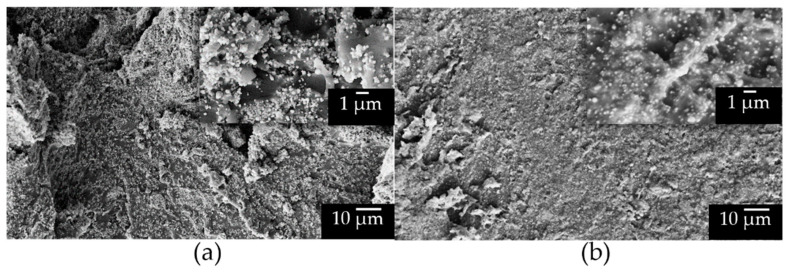
Cross-section SEM images, respectively, at 2000× magnification and 20,000× magnification (box close-up) of epoxy composites with 21 vol.% filler content. The particles employed are (**a**) BTH and (**b**) BTH_G.

SEM images of epoxy composites are shown in Figure 6 to highlight the different particle/matrix interfaces induced by the functionalization. The cross-sections analyzed correspond to the sample regions between the electrodes, subjected to the DC field. Figure 6a shows the epoxy composite loaded with bare BTH NPs at 21 vol.% filler content. The higher magnification highlights the poor filler–matrix interaction, showing well-defined particles. Using BTH_G particles (Figure 6b) at the same filler content leads to a more regular and smoother surface, and particles appear embedded in the epoxy matrix, thus confirming the positive effect of functionalization on the polymer-BT interface. Corresponding composites at 3.5 vol.% filler content present similar features. BTH_A particles behave similarly, leading to an improvement in the filler–matrix compatibility of the epoxy composite at 3.5 vol.% loading (Figure S2, see Supplementary Materials).

**Figure 7:**
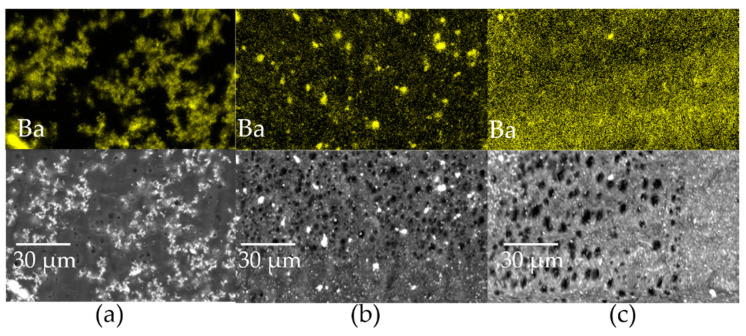
EDXS barium elemental maps and images of the reference areas of epoxy composites at 3.5 vol.% filler content loaded with (**a**) BTH, (**b**) BTH_G and (**c**) BTH_A. The holes observed in the reference areas are produced by prolonged exposure of the matrix to the high-energy electron beam during EDXS analysis.

EDXS elemental maps of Ba were recorded on sections subjected to the DC field of epoxy composites loaded at 3.5 vol.% in order to evaluate the dependence of particle dispersion on organosilane functionalization (Figure 7). With bare BTH NPs (Figure 7a), the composite presents regions rich in particles aggregated in clusters and others completely lacking fillers. Functionalization of BTH particles with the G silane (Figure 7b) produces an outcome with particles finely distributed, except for some clusters that appear slightly smaller compared to Figure 7a. On the contrary, BTH functionalization with A silane leads to a large improvement in particle dispersion in the epoxy matrix. The higher BTH_A dispersibility with respect to BTH_G is probably related to the different reactivity of the end-chain group of the two silanes, which leads consequently to the different strength of the filler–matrix interface. In the case of G, the epoxy ring is expected to react with the epoxy matrix, while the intensity of interactions of A-functionalized particles with the epoxy matrix is lower, resulting in less hindered particles and better dispersibility.

**Figure 8:**
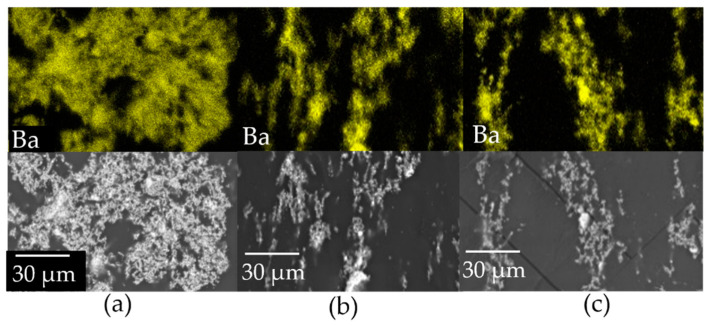
EDXS barium elemental maps and images of the reference areas of PDMS composites at 3.5 vol.% filler content loaded with (**a**) BTH, (**b**) BTH_G and (**c**) BTH_A. Holes observed in the epoxy composites are not found in PDMS composites because of their higher resistance to the high-energy electron beam.

SEM images of the cross-sections of PDMS composites show good matrix-filler continuity without noticeable differences, regardless of the employed particles. On the contrary, EDXS elemental maps of Ba highlight significant variations (particle alignment) due to the application of the DC field on composites produced with particles differing in size and surface modification (Figure 8). The alignment of BT particles in polymeric matrices has been already reported in the literature, and it was found to depend on several factors, such as particle size, aspect ratio, applied field, etc. [24]. In this work, with bare BTH particles (Figure 8a), large clusters (and depletion regions) are formed, giving evidence of a poor matrix-filler compatibility. At lower magnification, large clusters can be identified with partial segregation of the particles at the bottom of the sample, from which macrometric filaments extend vertically in the direction of the electric field, reaching in some cases the top face of the sample (Figure S3, see Supplementary Materials). The functionalization with the G silane of these particles (Figure 8b) further highlights this behavior. Filaments of a few tens of micrometers are observed, aligned along the direction of the applied external electric DC field. Employment of BTH particles functionalized with the A silane (Figure 8c) produces a result very similar to that of BTH_G particles (Figure 8b).

From these results, surface modification of the particles with G and A organosilanes clearly affects the distribution of BT particles in both epoxy and PDMS composites: in the first case, homogeneity in the distribution of the particles is enhanced, achieving the best results with the A silane; in the second case, the chemical modification allows one to increase the filler content, leading to the alignment of the particles along the poling direction and avoiding the formation of large, dense clusters and segregation of the particles at the bottom of the sample, with no detectable difference between the two silanes. There are almost no studies in the literature on the effect of silane coupling agents on BT NPs’ alignment. Todd and Shi [25] suggested that the presence of silane coupling agents could play a crucial role in modifying the molecular polarizability. Accordingly, it is worth noting that the results suggest the dependence of dispersibility and the alignment process on the chemical features of particle–matrix interfaces.

### 2.3. Dielectric Constant

**Figure 9:**
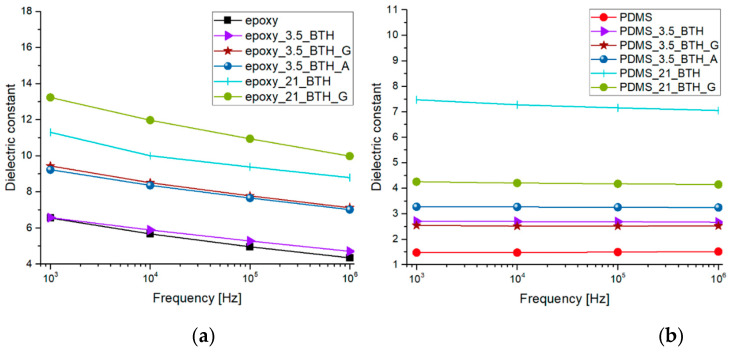
Values of dielectric constant in the range 1 kHz–1 MHz for epoxy-based composites (**a**) and PDMS-based composites (**b**) filled with BTH, BTH_G and BTH_A particles.

Figure 9 shows the results of dielectric constant measurements recorded on the different nanocomposites. Epoxy-based samples (Figure 9a) show a more pronounced decrease in the dielectric constant upon increasing the frequency, with respect to PDMS-based composites (Figure 9b). The decrease is due to the fact that dipoles are not able to follow fast orientation changes [26]. The higher polarity of epoxy with respect to silicone is responsible for the different behavior between the matrices, resulting in a decrease over the frequency of permittivity [26]. Furthermore, in the analyzed frequency range, the addition of barium titanate seems not to have effects on the decrease in dielectric constant over frequency. The dielectric properties of BT particles are demonstrated to be strongly dependent on the particle size. Wada et al. [27] reported high values of dielectric constant (up to 5000) with a BT particle size of 140 nm; our BTH particles present dimensions around 120 nm and this is highly beneficial to achieve a high dielectric constant. Accordingly, with BTH addition in epoxy-based samples (Figure 9a), the dielectric constant increases with a dependence on the particle load and functionalization [28]. Both at high and low filler loadings, a larger improvement is obtained with functionalized nanoparticles with respect to bare ones. Concerning PDMS samples, the situation is slightly different. Functionalization is not shown to improve the dielectric constant as much as in epoxy samples, and this is probably due to the different interaction of the silane coupling agent with the matrix. In fact, covalent bonds can be created between G-functionalized particles and the epoxy matrix through epoxy ring-opening reactions, while only weak interactions can take place between PDMS and NPs, resulting in a weaker particle–matrix interface compared to the epoxy-based system. However, the values of dielectric permittivity for PDMS samples at high BT loading values are particularly interesting when compared to the results reported in the literature [29], making the produced nanocomposites attractive for applications in the field of dielectric elastomers.

## 3. Materials and Methods

### 3.1. Materials

Commercial BT powders, with approx. diameter of 500 nm, were purchased from Acros Organics (Geel, Belgium, CAS 12047-27-7). A 30% aqueous solution of hydrogen peroxide H_2_O_2_ (Merck KGaA, Darmstadt, Germany, CAS 7722-84-1) and deionized water were used for the hydroxylation of the particles. The reagents employed for particles’ functionalization were 3-glycidyloxypropyltrimethoxysilane (G) (Merck KgaA, Darmstadt, Germany, CAS 2530-83-8), 2-[(acetoxy(polyethyleneoxy)propyl]triethoxysilane (A) (Gelest, Morrisville, PA, USA), triethoxysilyl-propoxy(polyethyleneoxy)dodecanoate (T) (Gelest, Morrisville, PA, USA, CAS1041420-54-5), toluene C_7_H_8_ (Honeywell, Charlotte, NC, USA, CAS 108-88-3), n-hexane C_6_H_14_ (Merck KgaA, Darmstadt, Germany, CAS 110-54-3), triethyl amine Et_3_N (TEA) (Merck KgaA, Darmstadt, Germany, CAS 121-44-8), acetone (CH_3_)_2_CO (Merck KGaA, Darmstadt, Germany, CAS 67-64-1). Commercial matrices for the nanocomposites were employed, namely a two-component epoxy system, Elan-Tron EC 251/W 242 (gently provided by Elantas Europe srl, Collecchio, Italy), and a two-component silicone rubber, Wacker Silgel 612 A/B (Wacker Chemie AG, Munich, Germany). Solvents such as acetone (CH_3_)_2_CO (Merck KGaA, Darmstadt, Germany, CAS 67-64-1) and pentane C_5_H_12_ (Merck KGaA, Darmstadt, Germany, CAS 109-66-0) were employed for the composites’ production. Kapton tape (Dupont, Wilmington, DE, USA) was employed for the poling process.

### 3.2. Commercial BT Particles’ Hydroxylation

One gram of BTC was dispersed in 100 mL of H_2_O_2_ by employing a 200 W bath sonicator (Ceia CP104, CEIA S.p.A., Arezzo, Italy) for 10 min, to break aggregates and better disperse the particles. The suspension was placed in a round-bottom flask equipped with a condenser and kept under stirring at 105 °C for two hours under N_2_ atmosphere and then in ambient atmosphere for different total times (4, 8, 24 and 48 h). Upon completing the reaction, the mixture was left to naturally cool down, and the particles were separated by centrifugation at 4000 rpm for 10 min. Then, powders were washed with deionized water three times, and dried in a vacuum overnight at 80 °C. The resulting particles were labeled BTC_4, BTC_8, BTC_24 and BTC_48, respectively.

### 3.3. BTC Particles’ Functionalization

Functionalization of hydroxylated BTC particles with 3-glycidyloxypropyltrimethoxysilane (G) was carried out in order to identify the hydroxylation time able to allow the highest functionalization grade. Here, 450 mg of hydroxylated particles was dispersed into 30 mL of toluene, and sonicated for 10 min. Then, 4.3 mL of G was slowly added under stirring (molar ratio G:BT = 10:1) and nitrogen flow, and the mixture was kept at 120 °C for two hours under N_2_ atmosphere and then in an ambient atmosphere for 22 h. Particles were separated by centrifugation at 4000 rpm for 10 min, washed three times with toluene and dried in a vacuum overnight at 80 °C. The functionalized particles were labeled BTC_4_G, BTC_8_G, BTC_24_G and BTC_48_G.

### 3.4. BTH Particles’ Functionalization

BTH particles were prepared by hydrothermal synthesis, as previously reported [21].

The optimized hydroxylation process (8 h) was adopted for increasing particles’ surface reactivity. Hydroxylation and functionalization of BTH particles with G were carried out with the same procedure used for BTC particles. Obtained particles were labeled BTH_G. Functionalization with 2-[(acetoxy(polyethyleneoxy)propyl]triethoxysilane (A) was performed, adapting a reported procedure [23]. First, 200 mg of hydroxylated particles was placed in a round-bottom flask with 16 mL of n-hexane and sonicated. Then, 0.28 mL of triethyl amine (weight ratio TEA/BT = 1) and 2.0 mL of A (molar ratio A/BT = 4.2) were added to the suspension, which was refluxed at 75 °C under N_2_ for 24 h. Recovered particles (BTH_A) were washed with acetone and dried, as reported above.

Functionalization with triethoxysilyl-propoxy(polyethyleneoxy)dodecanoate (T) was carried out similarly with a silane/BT molar ratio of 4.3, and the final particles (BTH_T) were washed with n-hexane before drying.

### 3.5. Composites’ Production

Composites were produced in epoxy resin and PDMS with five different types of particles: BTH, BTH_G, BTH_A. For each matrix/filler combination, composites with 3.5% and 21% volumetric filler content were produced, with a few exceptions: composites with A-functionalized particles were produced only at 3.5% content, and the bare BTH/PDMS composite at 21 vol.% was produced but with poor results as it was beyond the workability limit.

To produce composites, the particles were first dispersed in the liquid resin. In case of epoxy resin (Elan-Tron EC 251/W 242), resin (Elan-Tron EC 251) and hardener (Elan-Tron W 242), were dissolved in a 100/40 weight ratio in a small amount of acetone and then the particles were slowly added. The obtained mixture was stirred for 1 h, sonicated and further stirred until solvent evaporation. The mixture was then casted into 20-mm-diameter PTFE molds. One of these was equipped with a Kapton-tape-covered electrode to implement direct electric field application during cross-linking. The counter-mold was equipped with the same electrode and an O-ring was tightened around the upper mold close to the side of the electrode (Figure S4, see Supplementary Materials).

Once casted, molds were placed under a vacuum to remove residual solvent and air bubbles. The closed mold was then placed into a polystyrene chamber (Figure S5, see Supplementary Materials), and electrodes were connected to a high-voltage power supply (Spellman SL30, Spellman High Voltage Electronics Corporation). Temperature was set at 80 °C and voltage was increased so as to produce a direct electric field of 3 kV/mm, maintained for 90 min. After this time, heating was turned off to allow natural cooling to RT of the composites. Ten minutes after switching off the heating plate, the high-voltage supply was also switched off.

The procedure for producing PDMS-based composites (Wacker Silgel 612 A/B) was essentially the same: resin (Wacker Silgel 612 A) and hardener (Wacker Silgel 612 B) were dissolved in a weight ratio of 2:1 in a small amount of pentane. Mixing and degassing was as previously reported. The curing step was carried out with the same procedure but with a higher electric field (7 kV/mm). For reference, plain epoxy and PDMS samples were also produced.

### 3.6. Characterization of Nanoparticles and Nanocomposites

TGA-DTA curves were acquired with a Netzsch STA 409 thermobalance (Netzsch-Geraetebau GmbH, Selb, Germany) on hydroxylated and functionalized particles, respectively, from 30 to 800 °C, with a heating rate of 10 °C/min in flowing air. The weight losses attributed to the grafted silanes were calculated as the difference between the value at 800 °C and the value at the inflection point (350–400 °C).

Calculation of the grafting densities of the functionalized BTH samples was carried out by means of Equation (1) [23]:

(1) $$\sigma \;=\frac{m_{\mathrm{sil}}\cdot N_{A}}{\mathrm{MW}}\;\cdot \frac{1}{m_{\mathrm{BT}}\cdot \mathrm{SSA}}=\frac{X_{\mathrm{wt}}}{1-{\;X}_{\mathrm{wt}}}\;\cdot \frac{N_{A}}{\mathrm{MW}\cdot \mathrm{SSA}}$$

where $m_{\mathrm{sil}}$ is the organosilane weight loss [g], $N_{A}$ is the Avogadro number, MW is the molecular weight of the organosilane [g/mol], $m_{\mathrm{BT}}$ is the mass of employed particles [g], SSA is the specific surface area [nm^2^/g], $X_{\mathrm{wt}}$ is the percentual weight loss measured by TGA. With the hypothesis of uniform, spherical particles of diameter D, the specific surface area can be calculated with Equation (2):

(2) $$\mathrm{SSA}\;=\frac{{\pi \;D}^{2}}{\rho \;\cdot \;\frac{{\pi \;D}^{3}}{6}}=\frac{6}{\rho \;\cdot D}$$

where ρ is the density of the NPs, measured by employing a Micromeritics helium pycnometer, AccuPyc 1330 (Micromeritics Instrument Corp., Norcross, GA, USA).

Solid-state NMR experiments were performed with a Bruker 400WB spectrometer (Bruker Corp., Billerica, MA, USA) operating at a proton frequency of 400.13 MHz. Magic angle spinning (MAS) NMR spectra were acquired with a cross-polarization (CP) sequence with the following parameters: ^13^C frequency 100.48 MHz, contact time 2 ms, decoupling length 5.9 µs, recycle delay 3 s, 50 k scans (30 k for the quantitative analysis of particles functionalized with G); ^29^Si frequency 79.48 MHz, contact time 5 ms, decoupling length 6.3 µs, recycle delay 10 s, 20 k scans. Samples were packed in 4 mm zirconia rotors, which were spun at 8 kHz under air flow. Adamantane and Q_8_M_8_ were used as external secondary references. According to the common ^29^Si NMR notation, the Si species are labelled T^n^, where T represents SiO_3_C structural units, and n is the number of bridging oxygens. Preliminary acquisition of the NMR spectra of the silanes was carried out with the same instrument with proton-decoupled single pulse sequences under the following conditions: ^13^C frequency 100.48 MHz, π/2 pulse 7.2 µs, recycle delay 3 s, 1 k scans, decoupling length 80 µs; ^29^Si frequency 79.48 MHz, π/4 pulse 4.3 µs, decoupling length 5.2 µs, recycle delay 15 s, 64 scans. The liquid samples were added to a 4 mm zirconia rotor and analyzed without spinning. The quantitative analysis of the G-functionalized samples was carried out by weighting the 4 mm zirconia rotors before and after filling with the BT-functionalized particles and comparing the areas of the ^13^C resonances of methylene belonging to the propyl group of G with the same resonances of the ^13^C CPMAS spectrum of a known amount of a standard sample (STD, xerogel powder with a 33%mol of G); all the spectra were acquired and processed with the same parameters to allow the comparison.

SEM images of the nanoparticles were taken with a Carl Zeiss Gemini Supra 40 field emission scanning electron microscope (FE-SEM), (Carl Zeiss Microscopy GmbH, Jena, Germany), with an accelerating voltage of 8.0 kV at 80,000 magnification, using secondary electrons as the main signal. The same instrument employed for particles’ characterization was used to analyze the cross-sections of the nanocomposites, with an accelerating voltage between 7.5 kV and 10 kV, at 20,000 and 2000 magnifications. Before acquisition of the images, samples were metal-sputtered (Pt/Pd-80/20 alloy) with a Q150T coater (Quorum Technologies ltd, Lewes, UK). The cross-sections were characterized also by EDXS with a JEOL IT300 SEM equipped with an EDXS detector, operating at 20 kV.

DSC analyses were performed with a Mettler DSC30 (Mettler Toledo, Columbus, OH, USA), in a temperature range from −30 to 100 °C for epoxy-based samples and from −160 to 100 °C for PDMS-based samples, with a heating/cooling rate of 10 °C/min.

Dielectric permittivity was measured with an Agilent 4284A (20 Hz–1 MHz) precision LCR meter (Agilent Technologies, Santa Clara, CA, USA). The measurements were carried out with a probe signal of 1 V and a frequency sweep (1 kHz–10 kHz–100 kHz–1 MHz). The dielectric permittivity was indirectly evaluated from capacitance measurement by solving Equation (3) for ε_r_:

(3) C = ε0εrAd 

where C is the measured capacitance (in Farad, F), ε_0_ is the vacuum permittivity, ε_r_ is the relative permittivity of the material, A is the area of the sample and d is its thickness.

## 4. Conclusions

In conclusion, different organosilanes were successfully grafted on the surfaces of BT particles produced by sol-gel synthesis in hydrothermal conditions after an optimized hydroxylation process. The yield of the functionalization was evaluated both by solid-state NMR and TGA analyses, and the number of molecules per unit surface was also calculated, leading to values ranging from 10.6 to 3.3 molecules/nm^2^ depending on the type of silane. Moreover, 3-glycidyloxypropyltrimethoxysilane was proven to be more effective in surface functionalization with respect to 2-[(acetoxy(polyethyleneoxy)propyl]triethoxysilane and triethoxysilylpropoxy(polyethyleneoxy)dodecanoate, probably because of the different steric and electron-inductive effects of the organic chains. Functionalization was proven to be beneficial to enhance particles’ dispersibility and to reach higher filler content, thanks to the modified matrix-filler interface. Furthermore, particle alignment was observed in PDMS-based samples, and both the type of matrix and particle functionalization seem to play a key role in this process. Finally, preliminary dielectric studies were carried out by dielectric constant evaluation. According to these studies, particles were proven to enhance the value of the dielectric constant for composites, making them attractive as dielectric nanocomposites, particularly in the case of PDMS-based samples with high filler loading. A more complete electric and dielectric characterization of nanocomposites will be the subject of further studies.

## Figures and Tables

**Table 1:** Weight content of grafted 3-glycidyloxypropyltrimethoxysilane onto commercial BT particles hydroxylated at different reaction times, calculated from TGA and NMR data.

	BTC_4_G	BTC_8_G	BTC_24_G	BTC_48_G
TGA [wt.%]	0.6	2.1	1.4	1.8
NMR [wt.%]	0.8	2.4	1.7	2.0

## Data Availability

The data presented in this study are available on request from the corresponding author.

## Supplementary Materials

The following supporting information can be downloaded at: https://www.mdpi.com/article/10.3390/molecules27196499/s1, Figure S1: ^13^C CPMAS NMR spectra; Figure S2: SEM cross-section of epoxy 3.5% vol; Figure S3: Low-magnification EDXS of PDMS_3.5_BTH; Figure S4: Mold concept; Figure S5: Experimental apparatus; Table S1: Summary of DSC results.

## Sample Availability

Samples of the compounds are available on request from the corresponding author.
