# Supplementary material for: Barium Titanate Functionalization with Organosilanes: Effect on Particle Compatibility and Permittivity in Nanocomposites
Source: Molecules. 2022 Oct 1;27(19):6499. doi: 10.3390/molecules27196499 (PMC9572045; doi:10.3390/molecules27196499)
Supplement: Supplementary file 1 [file molecules-27-06499-s001.zip › molecules-1915101-supplementary.pdf]

molecules

MDPI

SUPPLEMENTARY MATERIAL

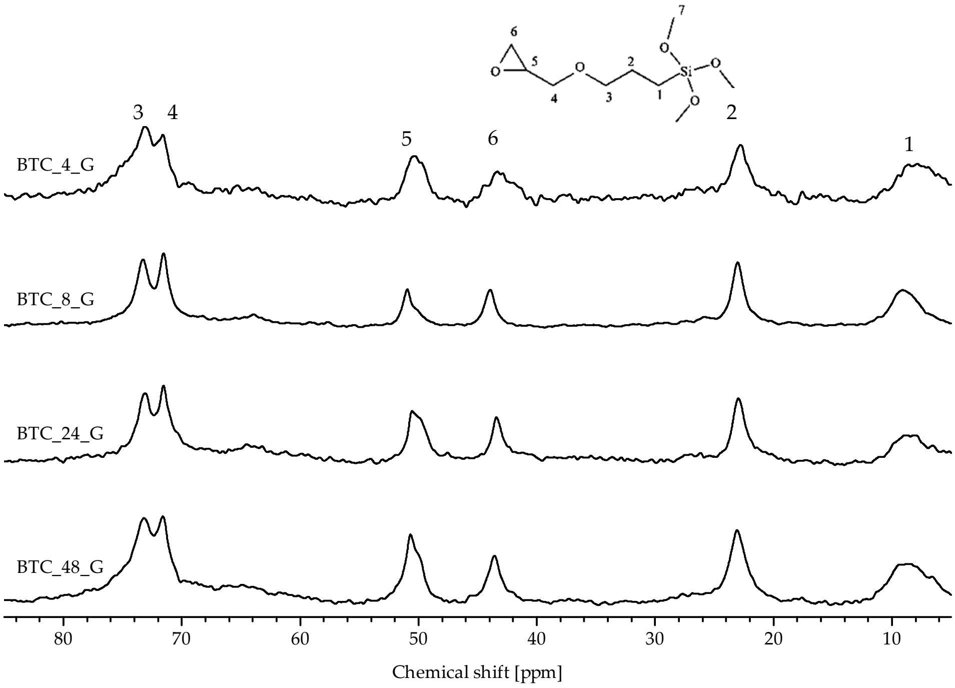

Figure S1. ^{13}C CPMAS NMR spectra of G-functionalized BTC particles hydroxylated for different time

Table S1. Results from DSC analysis on cured polymer-ceramic nanocomposites

Sample label	T_g [°C] (first heating)	T_m [°C]	ΔH_m [J/g]	T_{cc} [°C]	ΔH_{cc} [J/g]
**Epoxy**	9	-	-	-	-
**Epoxy_3.5_BTH**	6	-	-	-	-
**Epoxy_3.5_BTH_G**	8	-	-	-	-
**Epoxy_21_BTH**	8	-	-	-	-
**Epoxy_21_BTH_G**	26	-	-	-	-
**PDMS**	-125	-41	-28.24	-81	27.49
**PDMS_3.5_BTH**	-120	-37	-23.66	-84	24.14
**PDMS_3.5_BTH_G**	-124	-42	-23.52	-79	24.21
**PDMS_21_BTH**	-125	-42	-11.12	-82	10.73
**PDMS_21_BTH_G**	-127	-43	-13.67	-79	13.75

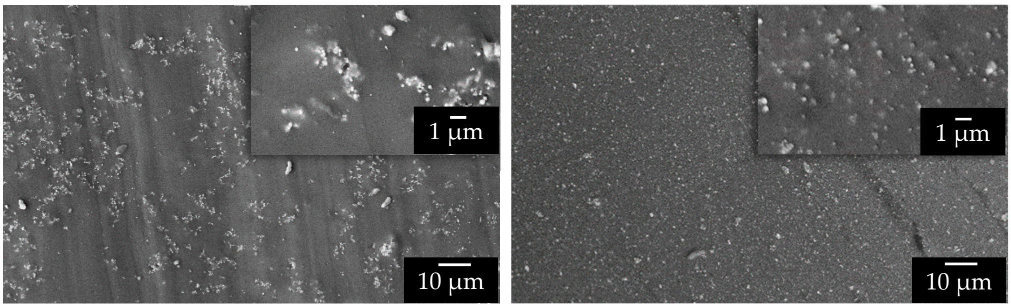

(**a**) (**b**)

Figure S2. Cross section SEM images of epoxy composites with 3.5 vol% filler content. The particles employed are (a) BTH and (b) BTH_G.

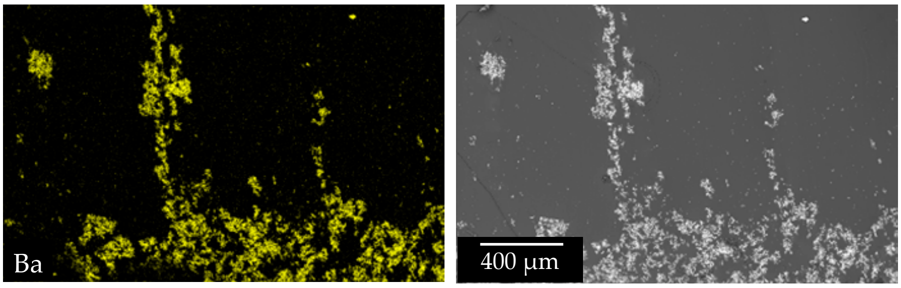

(**a**) (**b**)

Figure S3. EDXS barium elemental map (a) and image of the reference area (b) of the PDMS composite at 3.5 vol% filler content loaded with bare BTH particles.

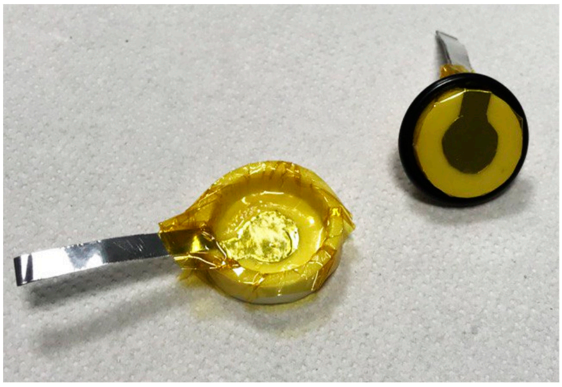

Figure S4. The modified mold used for poling during the cross-linking. Each part is equipped with an aluminum electrode and covered with a dielectric film. The positive mold has an O-ring tighten around to control the thickness of the final composite.

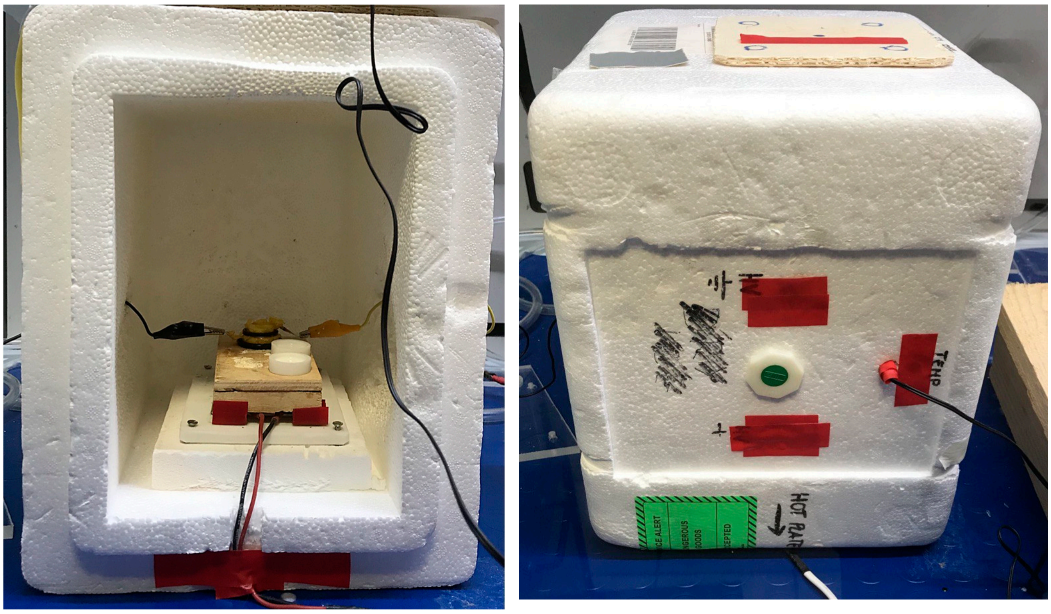

Figure S5. Experimental apparatus employed for combined poling/thermal curing of the nanocomposites; the filled molds are placed inside a polystyrene box equipped with the high voltage connectors and a heating plate controlled by a thermostat.
